# Uncovering the Influence of Operational Factors on Manufacturing Efficiency with Real Time Data

**DOI:** 10.1038/s41598-025-22776-8

**Published:** 2025-11-06

**Authors:** Pranita Bhosale, Sangeeta Jadhav

**Affiliations:** 1 Research Scholar, Dr. D. Y. Patil Institute of Technology, Pimpri-Chinchwad, Pune, Savitribai Phule Pune University (SPPU), India 0000-0001-6796-0872,; 2 Faculty, Department of Electronics and Telecommunication Engineering, Army Institute of Technology Dighi Hills, Pune, Savitribai Phule Pune University (SPPU), India; 3 Head of Department of IT Engineering, Army Institute of Technology, Dighi Hills, Pune, Savitribai Phule Pune University (SPPU), India 0000-0002-0610-0374,

**Keywords:** Predictive Maintenance, Plastic Extruder Machines, Temperature Sensors, Machine Learning (ML), Deep Learning (DL), Real-Time Data, Electrical and electronic engineering, Mechanical engineering

## Abstract

In modern manufacturing, OEE is a crucial metric, for assessing production efficiency. However, traditional OEE such as TOEE often missed out the key operational parameters-those that have the greatest influence on overall factory performance. This research provides comparative analysis using Traditional Overall Equipment Effectiveness (TOEE) and Modified Overall Equipment Effectiveness (MOEE). The research considers additional operational variables, which are ideal cycle time, downtime, roller performance and customer demand. This study seeks to provide a more dynamic and comprehensive view of manufacturing efficiency. The manufacturing production system is incorporating data in real time. The study looks into how machine performance data, shift schedules, the planned as well as unplanned downtime uncover hidden inefficiency. These findings align with MOEE’s ability to be more representative of how efficiently a company operates due to its accounting for more variables. This approach allows manufactures to put a finger on the pulse to recognize improvements area in real-time, to refine production processes and improve OEE. Leveraging true sustainability data: this paper shows activity recommendations when moving to a model that includes real time information with a view to refined performance metrics, such as the path to decision making in manufacturing operations. The findings highlight that MOEE provides a more comprehensive and accurate reflection of manufacturing efficiency compared to TOEE by incorporating real-time operational factors.

## 1. Introduction

Although OEE does not directly identify the reasons for machine inefficiencies, it classifies areas in need of improvement. Nakajima introduced the original OEE metric, where the availability element accounted for all machine downtime due to breakdowns, setups, adjustments, and other stoppages. However, this loss categorization merges all downtime events, which can obstruct the identification of specific losses, representing a significant limitation of OEE. Although loss classifications do not affect the final OEE value, it’s crucial to further define and refine these categories. This can be achieved without making the metrics too complex or deviating from its original calculations, as seen in other attempts. Like all business metrics, OEE has faced criticism in both literature and practice, and researchers have yet to comprehensively address these issues. This study aims to bridge the knowledge gap by exploring strengths, weaknesses, threats, and opportunities for further developing or restructuring the OEE metric to address its limitations. By further developing the OEE performance indicator, it can be enhanced to overcome criticisms while retaining its core calculation approach. Today’s ever-increasing demand for manufacturing facilities is to produce high-quality products. The high-quality products should meet challenging production timelines. These pressures are driven by factors such as increasing consumer demands, intensified industry competitiveness, and specific sector requirements, particularly in pharmaceuticals and medical devices. With the advent of industry 4.0 technologies, industries are moving closer to mass customization and the capability of producing batch sizes of one, further increasing the need for monitoring, improving, and maintaining productivity.

While Overall Equipment Effectiveness (OEE) has been a standard productivity performance metric for decades, it has never been more critical than it is today. Despite the introduction of the new technologies, systems, platforms and processes, OEE remains the best way to benchmark the productivity of manufacturing systems. It helps identify areas for improvement and provides a tool to objectively monitor progress towards enhanced productivity. Industry 4.0 and Smart Factory technologies enhance OEE’s effectiveness as manufacturing productivity measurement by improving the accuracy of its calculations. Key performance indicators like OEE may seem straightforward (simple), but their potential can be significantly enhanced by technologies under the broader Industrial Internet of Things (IIoT) umbrellas. However, linking IIoT and other technologies directly to OEE, from the machine level to the line and plant level, requires a strategic approach. Ultimately, the value of data collected is determined by how effectively it can be translated into actionable insights that address specific business problems.

OEE not only measures the effectiveness of production systems but also improves the durability of machinery through better maintenance practices. By applying OEE, production criteria are improved, and the industrial structure’s capacity is enhanced with limited capital investment. The upgrade of OEE is an essential initiative for accurate industrial advancement. Numerous efforts by researchers have sought to improve OEE to cover a broader perspective of production systems. The definition of OEE has evolved to include terms like Overall Factory Effectiveness (OFE), Overall Plant Effectiveness (OPE), Overall Throughput Effectiveness (OTE), Production Equipment Effectiveness (PEE), Overall Asset Effectiveness (OAE), and Total Equipment Effectiveness Performance (TEEP). A review of OEE literature highlights various methods for calculating OEE to meet the growing need for more holistic performance measures. However, these new performance metrics often shift away from original OEE calculation methodology.

Over time, OEE applications have evolved to meet industrial needs. Some researchers have modified the original formula, while others have proposed new formulas. Total Productive Maintenance (TPM) efforts focus on addressing major losses in the production process and fostering (encouraging) continuous, systematic calculations for improvement. OEE is a critical tool in driving performance improvement activities by diagnosing quality, productivity, and machine utilization issues, thereby reducing inherent manufacturing wastes.

The novelty of this work lies in the development of the Modified Overall Equipment Effectiveness (MOEE) framework that integrates real-time operational factors such as downtime, roller performance, ideal cycle time, and customer demand—parameters not considered in conventional TOEE studies. Unlike previous approaches, MOEE provides a dynamic and more accurate assessment of manufacturing efficiency by uncovering hidden inefficiencies through real-time data. This is significant for industry as it enables proactive decision-making and targeted process improvements, leading to enhanced productivity and sustainability.

## 2. Literature review

With the advent of Industry 4.0, a new maintenance paradigm has emerged, demanding the development of innovative methods, tools, and systems to meet new requirements. This shift is referred to as maintenance 4.0. However, there is lack of studies that assess the sustainability of current maintenance techniques in the context of Industry 4.0. Thus the problem addressed in this study is, how can we identify the most suitable maintenance technique to be developed to meet the demands of the factory of the future, which implements the concepts of I4.0?

The need for this research arises from the fact that each maintenance technique has its own unique features, advantages, and disadvantages. However, it is still unclear how these techniques will perform in an Industry 4.0 environment. This study aims to identify and select the most relevant maintenance techniques based on the maintenance features required by Industry 4.0. The goal is to provide maintenance professionals with insights that can guide the development of a proper maintenance strategy suited to the Industry 4.0 framework.

**Fig. 1:**
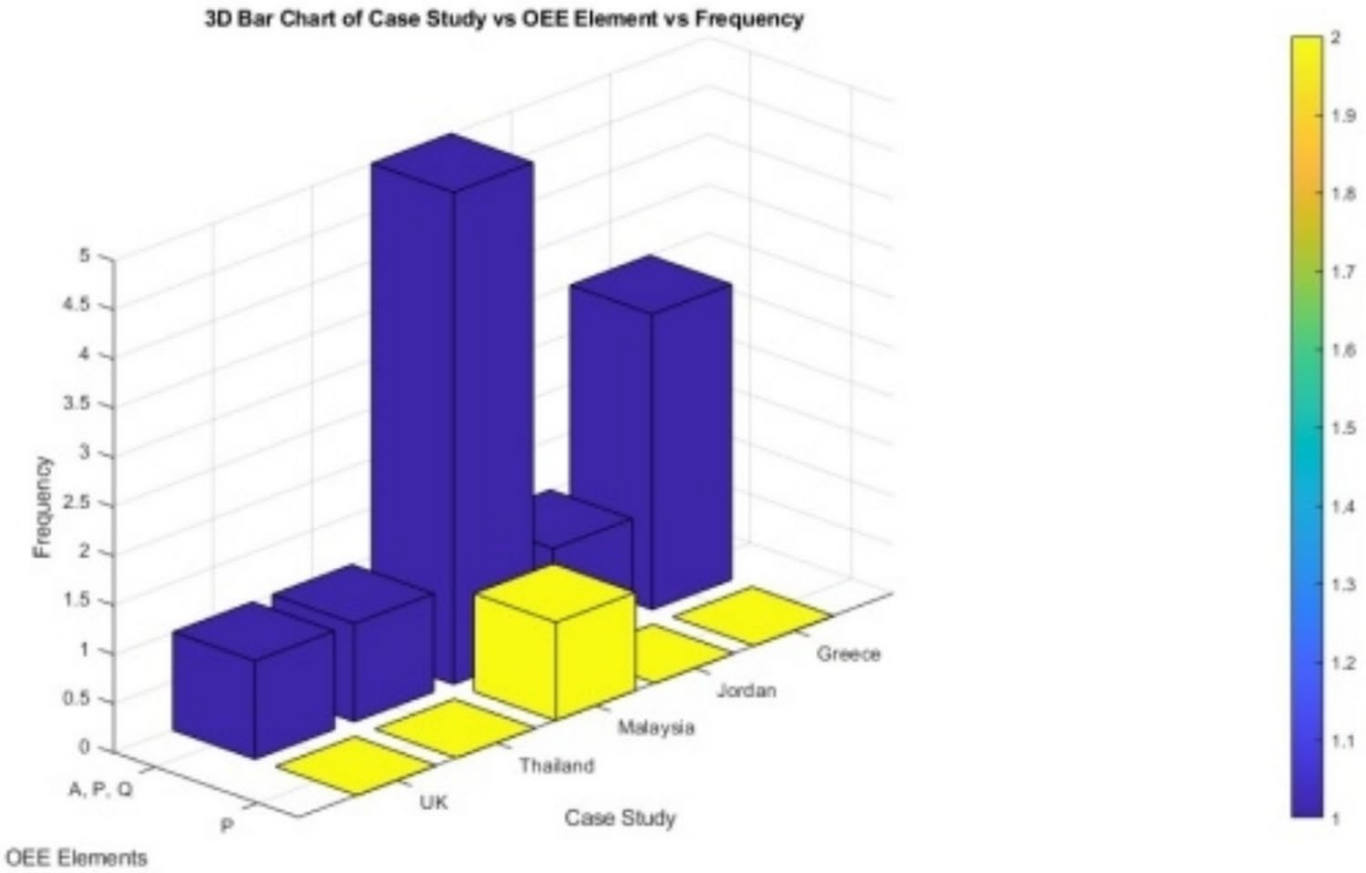
3D Bar Chart of Case Study Vs OEE (Overall Equipment Effectiveness)Elements Vs Frequency.

Figure 1 outline the total papers have been surveyed concerning OEE (Overall Equipment Effectiveness) elements: Availability (A), Performance (P), and Quality (Q). Based on the categories the paper explored improvement framework, guidelines and the use of OEE benchmarks. The survey results indicate that from 2000-2020, researchers focused on all OEE elements without providing improvement techniques.

From 25th to 28th August 2023, key inefficiencies were addressed with actions like scheduled servicing, training, and workflow improvements. MOEE offered more accurate and more reliable measure of efficiency than TOEE, especially in changing shifts, downtime, and maintenance. It considered factors like roller count, shift duration, and downtime impact to better forecast inefficiencies and reflect real-world performance. Below Table 1 elaborates the deeper literature review of all the references.

**Table 1:** Literature Review Summary on Industry 4.0 & Maintenance 4.0.

**Ref. No.**	**Author(s)**	**Focus area/technique**	**Contribution/Unique points**	**Gaps/observations**
^1^	Handbook of Techniques	Workplace practices	Maintaining employee discipline and good habits for safe environments	Not directly connected to I4.0 technologies
^2,4^	Chen L., Ziqiu K. et al.	AI & ML in manufacturing	Use of AI & ML to forecast manufacturing efficiency	Generalized approach; lacks case-specific deployment
^5^	Quoc N. et al.	AI-based production forecasting	Boosts OEE via predictive computations; uses deep learning	Focused on prediction, less on implementation framework
^3^	Puvanasvaran A. P. et al.	Enhanced OEE calculation	Introduced Usability and Human Factors; used MOST for waste visualization	Adds human-centric approach but less real-time integration
^6^	Rohan Thorat et al.	OEE & TPM in process industries	Case studies on Autonomous Maintenance for manual work	Limited to manual tasks; lacks I4.0 system context
^7^	L. A. E. Maalem et al.	OEE implementation	Focused on Availability element through case studies	Does not consider holistic OEE (P, Q)
^8^	Harris M. et al.	Industry 4.0 & maintenance	Literature review on I4.0 in maintenance	No new frameworks or experimental validation
^9^	M.H.M. Ahmed	Visualization of losses	Used SWOT analysis for loss visibility	Traditional tool not digitally integrated
^10,11^	Pranita B. et al.	Simulations for OEE	Proposed optimization using simulation & computational tools	Needs validation through real-world case studies
^12^	Ullah et al.	Power consumption-based OEE	Machine performance through energy data	Limited adoption in production lines
^13^	Moussa et al.	Sensor-based OEE	Applied OEE to full line via sensors; aligns with I4.0 model	Needs cost-benefit analysis
^14,20^	Zineb M., Bambang S.	Real-time OEE monitoring	Enabled tracking via real-time dashboards	No predictive or AI analytics mentioned
^15^	Almashaqbeh et al.	Framework for plastic industry	Loss identification using Pareto Analysis	No AI or IoT integration
^16^	Aziana et al.	Lean manufacturing 4.0	Trends, applications, and impacts of Lean in I4.0	Conceptual; lacks case data
^17^	Laura L. et al.	Lean TPM + I4.0	Integrated lean TPM with analytics tools	Promising but early-stage
^18^	Manojdeep S. J.	Digital SMED	Improved setup times and OEE via Digital SMED	Needs generalization to other areas
^19^	Pranita B. et al.	ML/DL for predictive maintenance	PNN showed 99.7% accuracy on real-time dataset	Needs real-world deployment validation

## 3. Methodology

The dataset used in this study by Pranita B. et al.^19^ is sourced from ‘Radhan Plastics’, a company established in 2008 as a Partnership Firm. ‘Radhan Plastics’ specializes in manufacturing films, tubing, rolls, bags, and covers from materials such as EVA (Ethyl Vinyl Acetate), VCI (Vapour Corrosion Inhibitor), Bubble, LDPE (Low-Density Polyethylene). Their products are available in various designs, colours, sizes, and shapes to meet diverse customer needs. These products find extensive application in: Rubber compounding, Pharmaceuticals, Food industry, Agriculture, Industrial packaging, Auto component/spares packing, and other industrial packaging applications. The company’s manufacturing facility is located in the area of ‘Pirangut’, near Pune, India. Their clientele spans across India, including cities such as Roorkee, Mumbai, Jammu, Bangalore, and Hyderabad.

Plastic Extrusion is a high-volume manufacturing process. In this process, raw plastic or plastic pellets/granules is liquefied and shaped into a uniform shape. The process begins by pouring plastic material i.e. raw input into a hopper, which leads into the extruder barrel. Inside the barrel, the material is gradually melted; the molten plastic is pushed through a die. The die gives the plastic its final shape, which hardens as it solidifies during cooling.

### 3.1 Data acquisition

The dataset consists of individual file (CSV file) that is 1 second temperature signal per row per second. The sampling frequency is 1Hz. There are four temperature sensor used for continuous temperature monitoring. Mainly RTD (Resistance Temperature Detector) is used for continuous temperature monitoring and one more sensors that are EM Proximity sensor used for counting of the final product at the end. This research study is completely dependent on Temperature data set only, no other extra sensor used to compute MOEE.

RTD sensors, specifically Pt100, are commonly used for temperature measurement in industrial applications, because of its High accuracy, wide temperature range for Pt100 is −200°C to 850 °C, good stability. The ID12-3002NA is a specific model of proximity sensors used for detecting the presence of objects within a defined range of 2–10 cm without any physical contact. It can sense objects within this distance from the sensor’s surface. The temperature data file contains 3 main groups of data as:

1. Total Number of Sensors: 04
2. Data Recorded: 3 Months per minute
3. Total count of no. of rows in csv file: 263146

Normal temperature range of Plastic Extruder machine is between 200º C and 220 º C, considered this state as ‘Warning 0’ where Machine is working correctly. Another state with machine temperature is in between 180 º C and 199 º C then considered this state as ‘Warning 1’ where Machine is working correctly but need to monitor, lastly when Temperature less than 180 º C or greater than 220 º C, considered this state as ‘Warning 2’, which is a High Alert where Machine is not working correctly and corrective action need to be taken urgently. Alert gets created when there is a fluctuation in temperature which leads to no. of waste products. And exactly during such conditions the parameters of MOEE plays their vital role to compute losses with respect to A, P, Q, U and C parameters.

The ‘Performance’ parameter, it measures how fast the machine was really working compared to its best it could possibly do. All four variables Total Output, Ideal Cycle Time, Operating Time, and Waste Time are dependent parameters, used to calculate ‘P’ performance. Among these, Waste Time is closely connected to temperature. Any fluctuation in temperature can negatively affect the machine, leading to increased waste time and lower performance. It is important to track each of these variables accurately to ensure reliable and meaningful performance metrics.

**Fig. 2:**
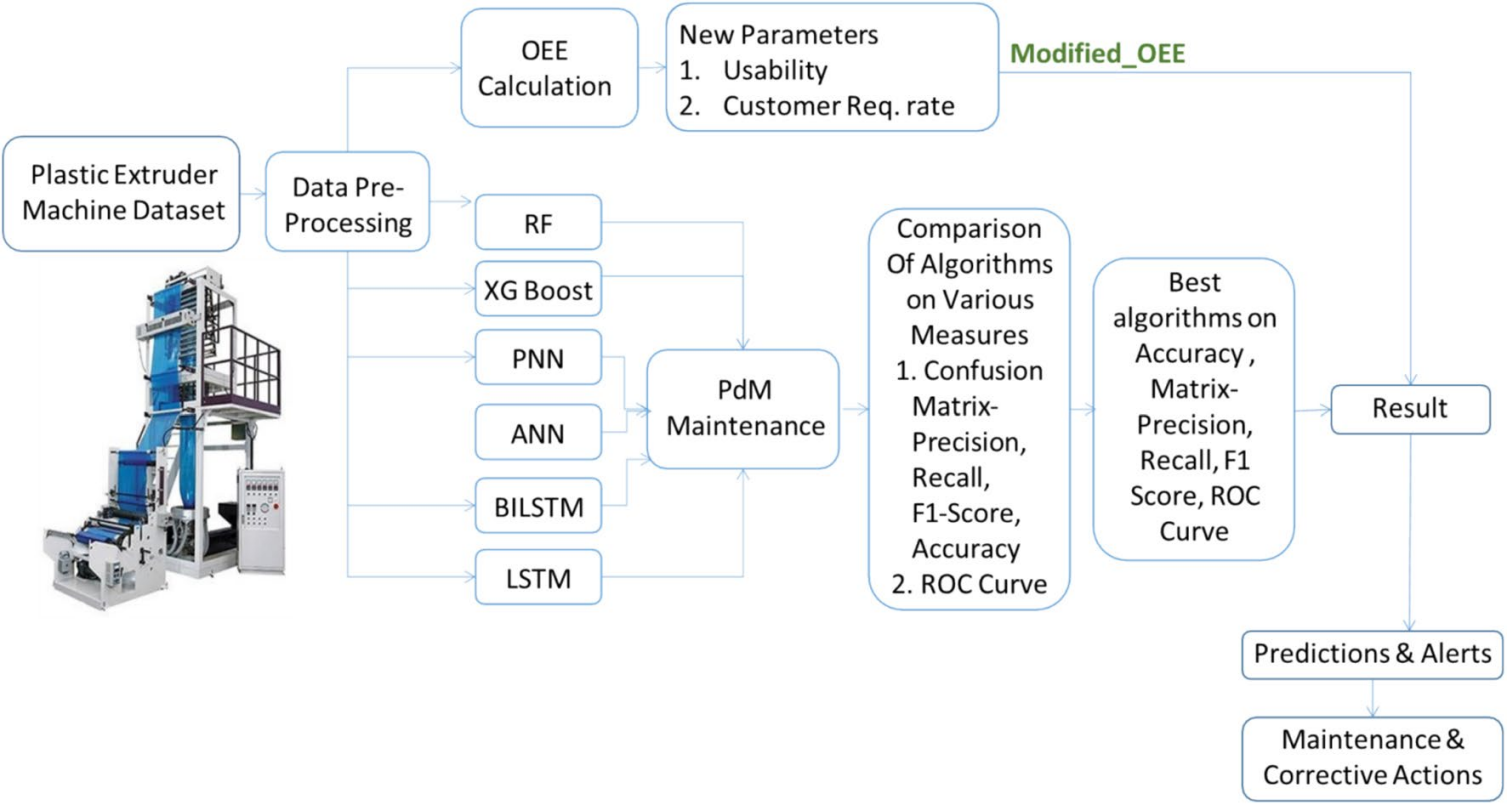
Overall Block Diagram of the Proposed System Architecture.

Figure 2 outlines the overall block diagram of the proposed system. It consists of two Parts, this study limited to Part ‘A’ only i.e. Modified OEE. The study provides in-depth understanding of “How TOEE and MOEE improve Overall Equipment Effectiveness (OEE)”. Their major role plays in modern manufacturing processes.

Table 2, 3 & 4 outlines the recorded data which consists of various columns that capture different aspects of machinery activities; production shifts/shift timings, and downtime/machine stoppages. It is used for productivity assessment, particularly in OEE (Overall Equipment Effectiveness). It also helps in machine stoppage evaluation and other production-related analyses. The recorded data appears to be sequential. Timestamps capture the start and end of various operational events like shifts or downtime. These recorded times or event logs are typically used to measure elapsed times for various workflows. They are crucial to evaluating the efficiency of manufacturing activities.

**Table 2:** Dataset for Further Processing.

**recid**	**jobcardid**	**machinecode**	**rollerid**	**rollcount**	**assessedlength**	**assessedkg**	**coreweight**	**rollweight**	**actualkg**	**tags**	**comment**	**tscreated**	tslastupdated
1146	3394	ex04	A	7	0.871	18.8	0.97	\N	\N	from rt log trigger	RUNNING	12/24/2023 3:59	12/24/2023 5:11
1145	3398	ex06	B	2	2.35	57.9	1.43	\N	\N	from rt log trigger	RUNNING	12/24/2023 3:20	12/24/2023 5:11
1144	3398	ex06	A	1	0.426	10.4	1.24	56.7	55.5 kg	from rt log trigger	COMPLETED	12/24/2023 1:40	12/24/2023 4:19
1143	3375	ex05	A	1	2.97	433	2.15	\N	\N	from rt log trigger	RUNNING	12/24/2023 1:21	12/24/2023 5:11
1142	3369	ex05	B	2	\N	\N	2.65	\N	\N	\N	Loaded	12/24/2023 1:03	12/24/2023 1:03
1141	3369	ex05	A	1	0.221	36.1	2.61	19.1	16.4 kg	from rt log trigger	COMPLETED	12/24/2023 1:02	12/24/2023 1:21
1140	3394	ex04	A	6	2.32	50.7	1.11	25.2	24.1 kg	from rt log trigger	COMPLETED	12/24/2023 0:47	12/24/2023 3:59
1139	3392	ex06	A	2	0.589	38.5	2.39	39.7	37.3 kg	from rt log trigger	COMPLETED	12/24/2023 0:35	12/24/2023 1:37
1138	3367	ex05	A	7	0.243	92.8	3.16	\N	\N	from rt log trigger	RUNNING	12/24/2023 0:28	12/24/2023 1:02
1137	3367	ex05	B	6	\N	\N	2.8	\N	\N	\N	Loaded	12/23/2023 23:52	12/23/2023 23:52
1136	3367	ex05	A	5	0.794	318	2.64	48.5	45.9 kg	from rt log trigger	COMPLETED	12/23/2023 23:14	12/24/2023 0:27
1135	3367	ex05	B	4	\N	\N	2.84	\N	\N	\N	Loaded	12/23/2023 22:37	12/23/2023 22:37
1134	3392	ex06	B	1	0.397	13.7	2.43	38	35.6 kg	from rt log trigger	COMPLETED	12/23/2023 22:30	12/24/2023 1:58
1133	3394	ex04	A	5	1.55	33.9	0.95	22.1	21.1 kg	from rt log trigger	COMPLETED	12/23/2023 22:14	12/24/2023 0:46
1132	3384	ex06	A	1	0.903	59.1	1.58	51.1	49.5 kg	from rt log trigger	COMPLETED	12/23/2023 22:05	12/23/2023 23:45
1131	3408	ex03	B	1	2.14	223	1.91	\N	\N	from rt log trigger	RUNNING	12/23/2023 21:43	12/23/2023 23:05
1130	3367	ex05	B	3	\N	\N	4.29	\N	\N	\N	Loaded	12/23/2023 21:42	12/23/2023 21:42
1129	3367	ex05	A	2	0.775	310	3.11	40.5	37.4 kg	from rt log trigger	COMPLETED	12/23/2023 21:29	12/23/2023 23:13
1128	3367	ex05	B	1	\N	\N	3.23	\N	\N	\N	Loaded	12/23/2023 20:44	12/23/2023 20:44
1127	3390	ex06	B	4	1.64	65.6	1.12	\N	\N	from rt log trigger	RUNNING	12/23/2023 19:45	12/23/2023 22:21
1126	3349	ex05	B	15	\N	\N	3	\N	\N	\N	Loaded	12/23/2023 19:40	12/23/2023 19:40
1125	3404	ex03	B	5	3.42	327	1.85	54.6	52.8 kg	from rt log trigger	COMPLETED	12/23/2023 19:18	12/23/2023 21:42
1124	3370	ex01	A	10	0.305	11.1	1.31	62.2	60.9 kg	from rt log trigger	COMPLETED	12/23/2023 19:05	12/23/2023 22:55
1123	3349	ex05	A	14	0.0916	36.6	3.1	36.4	33.3 kg	from rt log trigger	COMPLETED	12/23/2023 18:45	12/23/2023 21:28

**Table 3:** Data Extraction for Further Analysis and Calculations.

**date**	**shift_in_minutes (Min)**	**Planned_downtime_tea_lunch_dinner (Min)**	**num_rollers**	**num_good_rollers**	**num_rollers_waste**	**ideal_cycle_time (Min)**	**Customer_demand**
16-Aug-23	59.833333	15	5	5	0	24	10
21-Aug-23	242.26667	60	3	3	0	24	8
22-Aug-23	406.53333	120	8	8	0	24	13
25-Aug-23	688.61667	120	13	13	0	24	18
26-Aug-23	418.23333	120	12	11	1	24	17
27-Aug-23	853.5	120	26	26	0	24	31
28-Aug-23	691.01667	120	9	9	0	24	14
5-Sep-23	135.16667	15	5	1	4	24	10
13-Sep-23	12.166667	15	2	2	0	24	7
15-Sep-23	358.98333	60	8	8	0	24	13
16-Sep-23	621.61667	120	35	35	0	24	40
17-Sep-23	449.95	120	37	36	1	24	42
18-Sep-23	705.95	120	43	41	2	24	48
20-Sep-23	787.96667	120	34	33	1	24	39
22-Sep-23	919.3	120	33	32	1	24	38
23-Sep-23	561.58333	120	12	12	0	24	17
27-Sep-23	544.46667	120	22	20	2	24	27
28-Sep-23	247.08333	60	7	7	0	24	12
29-Sep-23	227.86667	60	6	5	1	24	11
30-Sep-23	185.06667	60	4	4	0	24	9

**Table 4:** Employee Shift Details and Downtime Allocation (including Breaks).

Shift (Hr)	Shift (Min)	Planned downtime (Min) (Tea+Lunch+Dinner)
3	< 180	15
6	181–360	60
16	361–990	120
22	991–1320	240

**Fig. 3:**
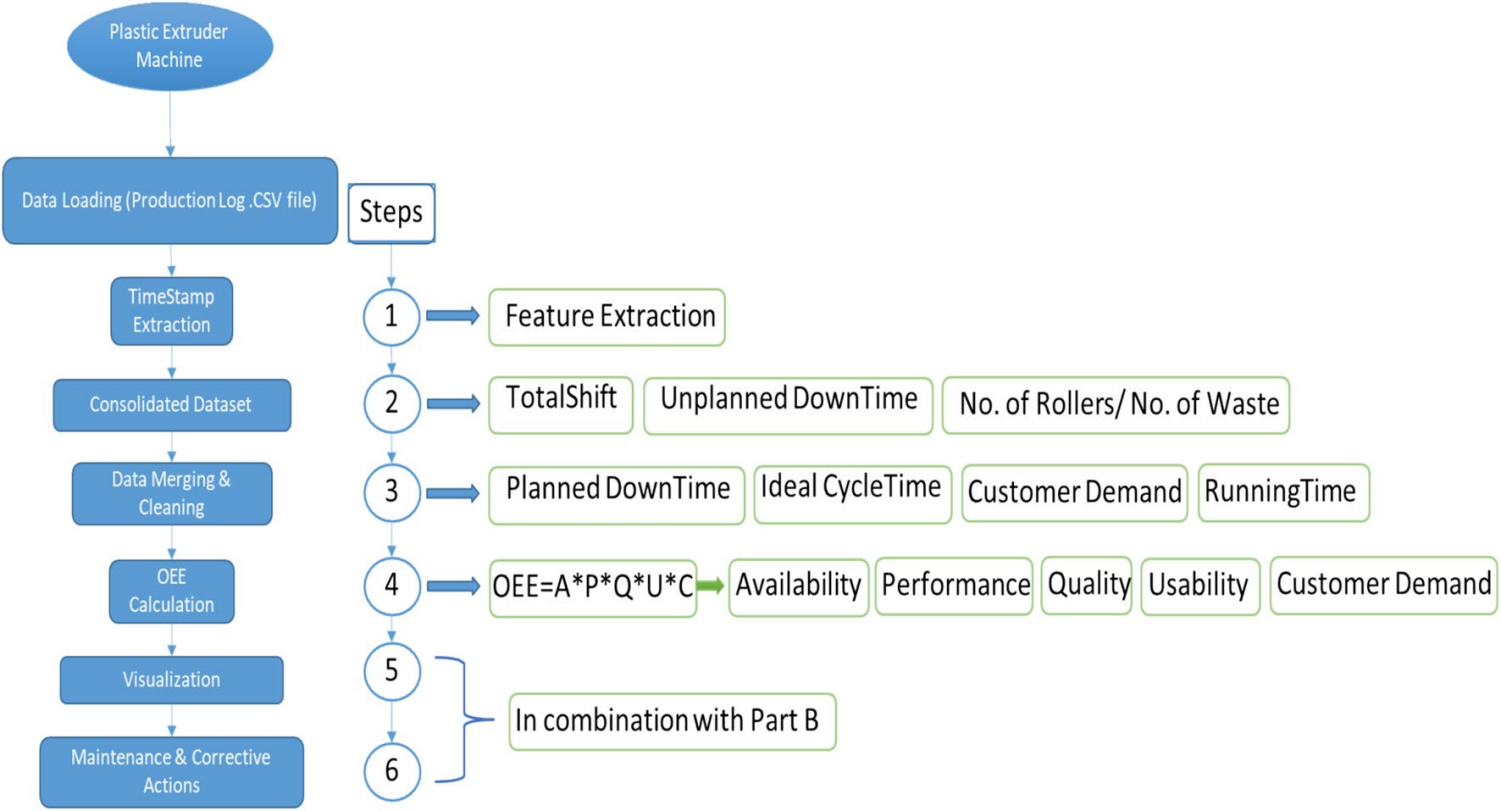
Flow Diagram of the Proposed System Architecture.

**Fig. 4:**
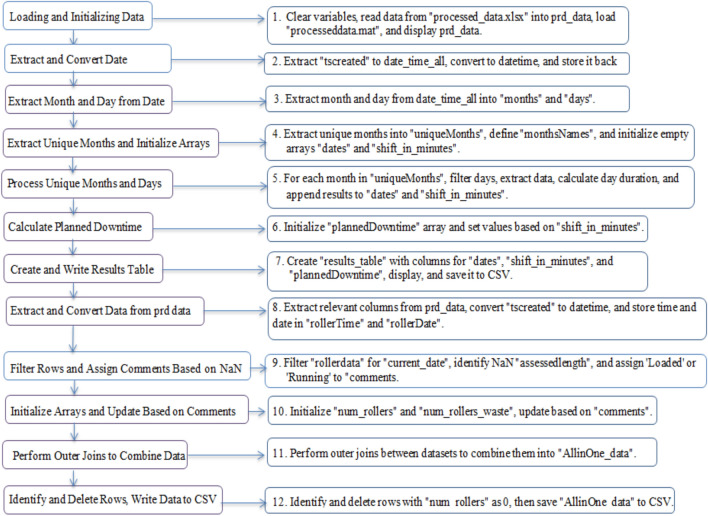
Program Execution Flow Diagram - Process Overview.

Figure 3 & 4 outline the dataset, includes step by step execution where timestamps that are formatted as dd-MM-yyyy HH\: mm and the code performs date-time conversion to make it compatible for analysis. The datetime function in MATLAB is used to convert these timestamps into a datetime object, which allows convenient extraction of months, days, and hours for time-based analysis. The dataset contains shift data, and the code extracts information related to the duration of each shift by comparing the start and end times within the same day. This is critical (vital) for assessing performance across different shifts.

Planned downtime is categorized based on the duration of shifts. Different ranges of shift durations are assigned specific planned downtime values (15, 60, 120, or 240 minutes). This categorization helps distinguish between planned and unplanned downtimes and is used in performance metrics like OEE. For certain columns (like assessed length), missing or NaN values are handled by classifying the equipment as either “Running” or “Loaded”.

This ensures that even incomplete data can be classified and analyzed effectively. The dataset from multiple sources (shift data, roller data, and unplanned downtime) is merged using the outerjoin function. This enables the integration of data from different sources. The merging is done based on common dates. The consolidated information provides a comprehensive view of operations. The analysis considers various factors such as operational effectiveness, production halts and shift durations.

### 3.2 Data pre-processing

Data sanitization and preprocessing is an essential in data analysis. It ensures that the data is error-free, standardized, and suitable for evaluation. The process involves several steps. These tasks include addressing null entries, standardizing timestamps, eliminating redundant records and transforming data structures. Below is an in-depth explanation of the data cleaning and refinement techniques implemented in MATLAB code.

#### 3.2.1 Missing data handling

- Missing Values (NaNs):

- This explicitly checks for missing values in the assessedlength column.
- Pseudocode:

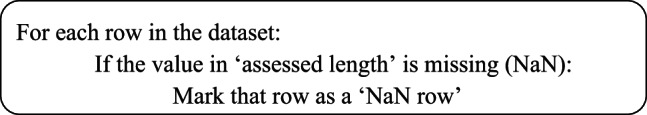

b) Handling of missing data:

- Rows with missing assessedlength are labeled as ‘Loaded’, others as ‘Running’.
- This is a domain-specific strategy to make use of incomplete data without deleting it.
- Pseudocode:

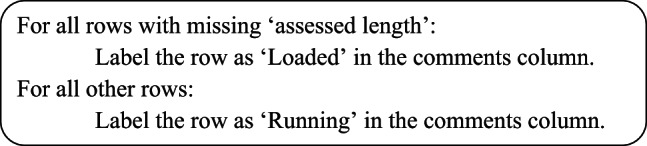

c) Missing data:

- Missing values are still used meaningfully in later calculations, so they are not discarded but integrated into analysis.
- Pseudocode:

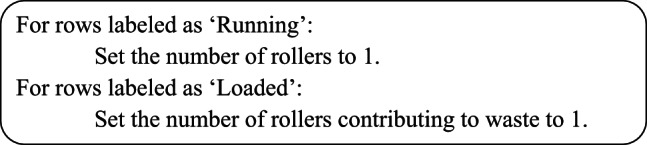


#### 3.2.2 Outlier Detection/Handling

- This is a fixed threshold approach:

- Shift durations outside expected ranges are marked as NaN, which may represent outlier filtering for abnormal shift values.
- Pseudocode:

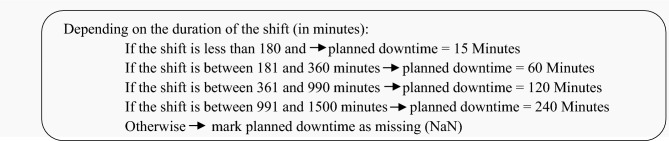


#### 3.2.3 Date-Time Processing and Aggregation:

- These steps are not strictly outlier/missing handling but are essential for time-based analysis
- Extracted and computed shift durations in minutes.
- Used for calculating planned downtimes.
- Pseudocode:

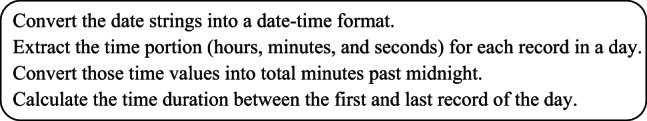


#### 3.2.4 Data Cleaning and Filtering

- This is a critical data cleaning step, removing rows with no production activity, which has skew metrics like OEE.
- Pseudocode:

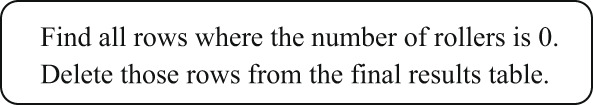


#### 3.2.5 Merging Multiple Datasets

- Used outer joins, which retained unmatched records with NaNs.
- Pseudocode:

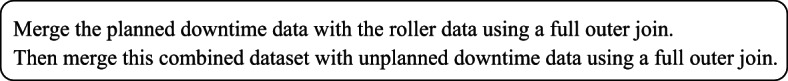


### 3.3 Formula for OEE and MOEE Calculation

**Fig. 5:**
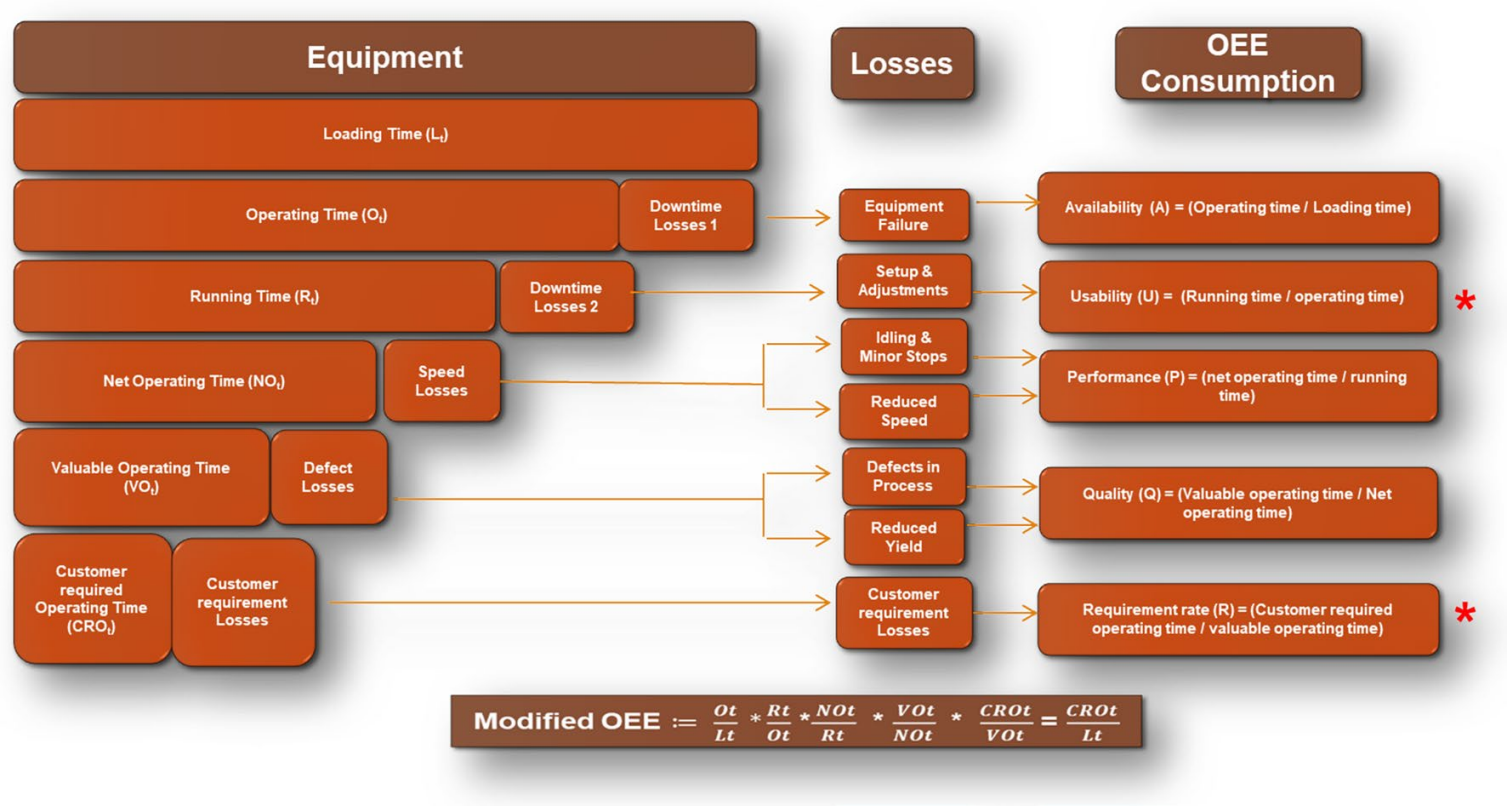
Understanding Modified OEE with Loss Contributions.

Figure 5 outlines the parameters of OEE and their respective losses. Here in this section definition of each parameter is discussed with their formulae. The dataset is primarily used to calculate performance metrics such as Overall Equipment Effectiveness (OEE), which is an industry-standard measure of machine performance. The dataset includes data related to:

- **Availability**: The proportion of time the equipment is available to run versus being down.
- **Performance**: The efficiency of the equipment while it is running.
- **Quality**: The proportion of good items produced.

By evaluating the data on shift durations, non-operational periods, and machine condition, it is possible to calculate the OEE (efficiency metric). This helps in making well-founded decisions regarding equipment maintenance, efficiency improvement, and resource.

#### 3.3.1 TOEE calculation

OEE (Overall Equipment Effectiveness) is a key metric used to assess the effectiveness of a manufacturing process. It consists of three main factors: Availability, Performance, and Quality.

**1. Availability (A)**: This factor measures the time during which the equipment is available for production. It takes into account both planned and unplanned downtime.

1 $$\text{Availability}= \frac{(\text{Total Available Time}-\text{Planned Downtime}-\text{Breakdown})}{\text{Total Available Time}}$$

**2. Performance (P)**: Performance assesses how quickly the machine is operating compared to its ideal cycle time.

2 $$\text{Performance}=\frac{\left(\text{total output }*\text{ ideal cycle time}\right)}{\text{operating time }-\text{ total waste time}}$$

**3. Quality (Q)**: This factor measures the proportion of good units produced versus total output.

3 $$\text{Quality }=\frac{\left(\text{Total Good Units }\right)}{\text{Total Output}}$$

#### 3.3.2 MOEE calculation

MOEE (Modified OEE) is the advanced version of OEE, considering additional factors like usability and customer requirements. Here’s how we compute it:

**1. Usability (U)**: Usability measures the percentage of the total operating time that the equipment is running.

4 $$\text{Usability }=\text{ Running Time}/\text{Operating Time}$$

**2. Customer requirement rate (C)**: This is a measure of how well the production process is meeting the customer demand in terms of time.

5 $$\text{Customer Requirement Rate}=\frac{(\text{Customer Demand}\times \text{Ideal Cycle Time})}{\text{Valuable Operating Time}}$$

**Fig. 6:**
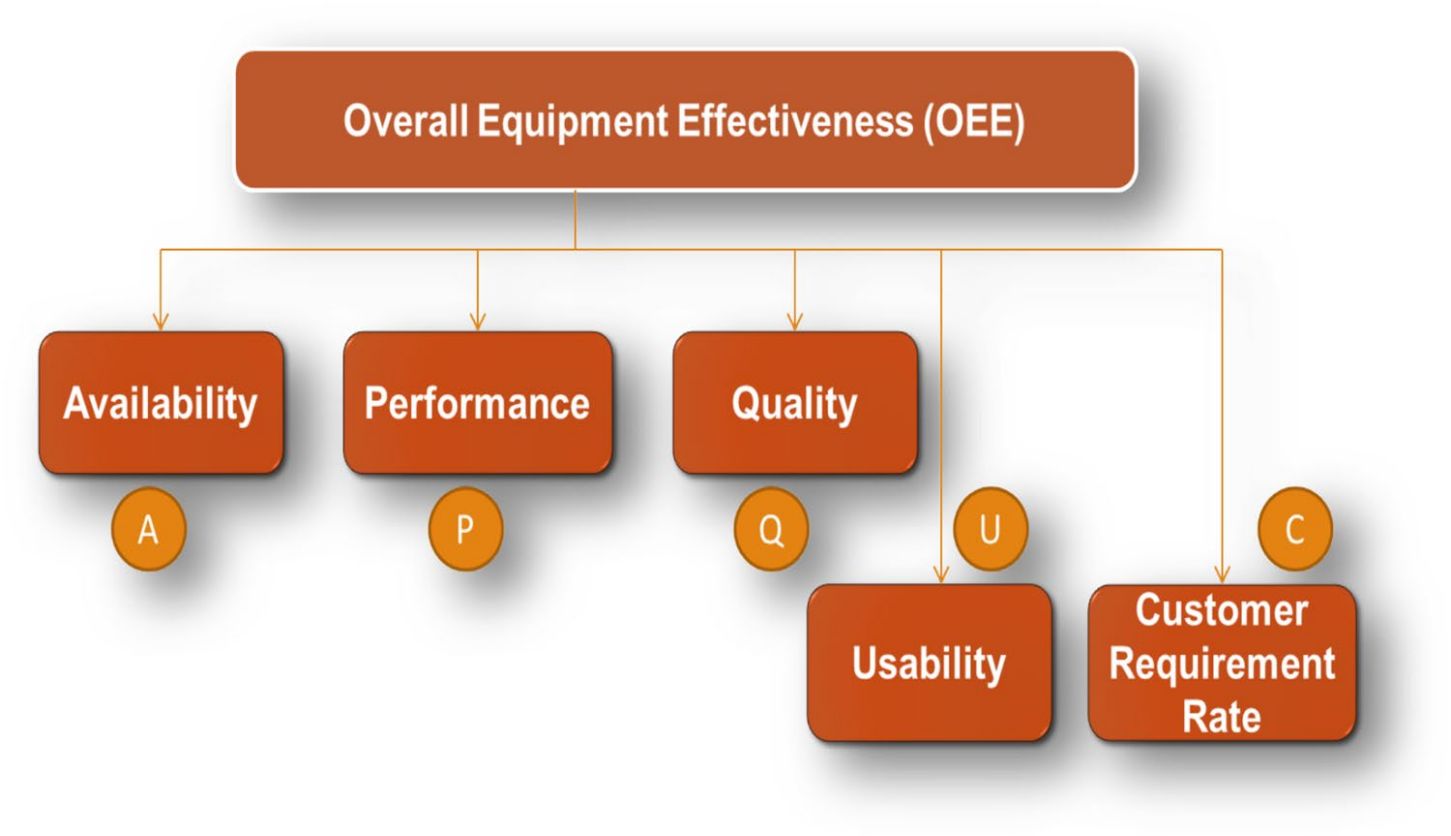
Modified OEE Parameters - A Comprehensive Breakdown.

Figure 6 outlines the **MOEE** includes the factors of Availability (A), Performance (P), Quality (Q), Usability (U), and Customer Requirement Rate (C), the formula for **MOEE** can be expressed as:

6 $$MOEE=A*P*Q*U*C$$

Where:

**A** = Availability, **P** = Performance, **Q** = Quality, **U** = Usability, **C** = Customer Requirement Rate.

## 4. Results and discussion

This research is currently restricted to data from a temperature sensor within a single plastics manufacturing company. While efforts were made to obtain sensor data from multiple industries, real-time data sharing poses significant barriers due to confidentiality concerns. Despite approaching several organizations, access to proprietary sensor data remains unavailable.

Although sample datasets are publicly available online and have been used in previous publications, this research work specifically focused on real-time sensor data and the application of the proposed Modified Overall Equipment Effectiveness (MOEE) methodology to that data. This is a significant advancement, as it grounds the method in real operational conditions.

Regarding generalizability, the MOEE framework may require tuning in preprocessing steps depending on the nature of the data, particularly across sectors with different process types (e.g., continuous vs. discrete manufacturing). However, the core MOEE methodology itself is designed to be a versatile and adaptable approach to calculating equipment efficiency across various manufacturing settings.

**Fig. 7:**
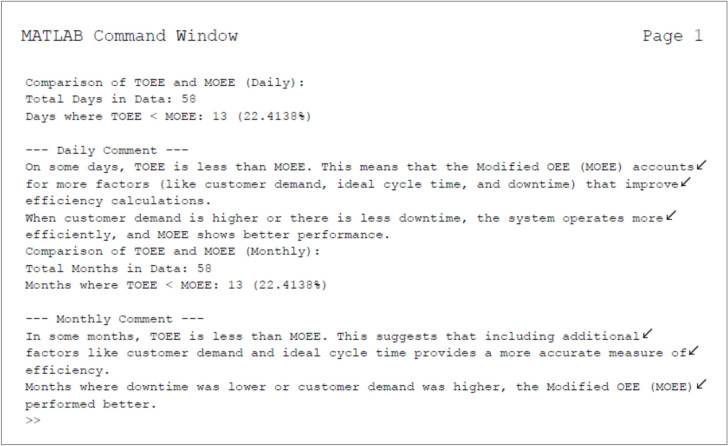
MATLAB Command Window Output - Key Results and Insights.

Figure 7: MATLAB result window presents the daily as well monthly findings of TOEE and MOEE over the evaluated period to identify broader efficiency trends. Both TOEE and MOEE are essential for assessing operational efficiency in manufacturing. MOEE includes additional factors that may cause variations compared to TOEE. A statistical summary of daily performance reveals cases where: TOEE was lower than MOEE. And this suggests that modifications in MOEE affect efficiency calculations. MOEE accounts for factors like Downtime and Operational losses.

**Fig. 8:**
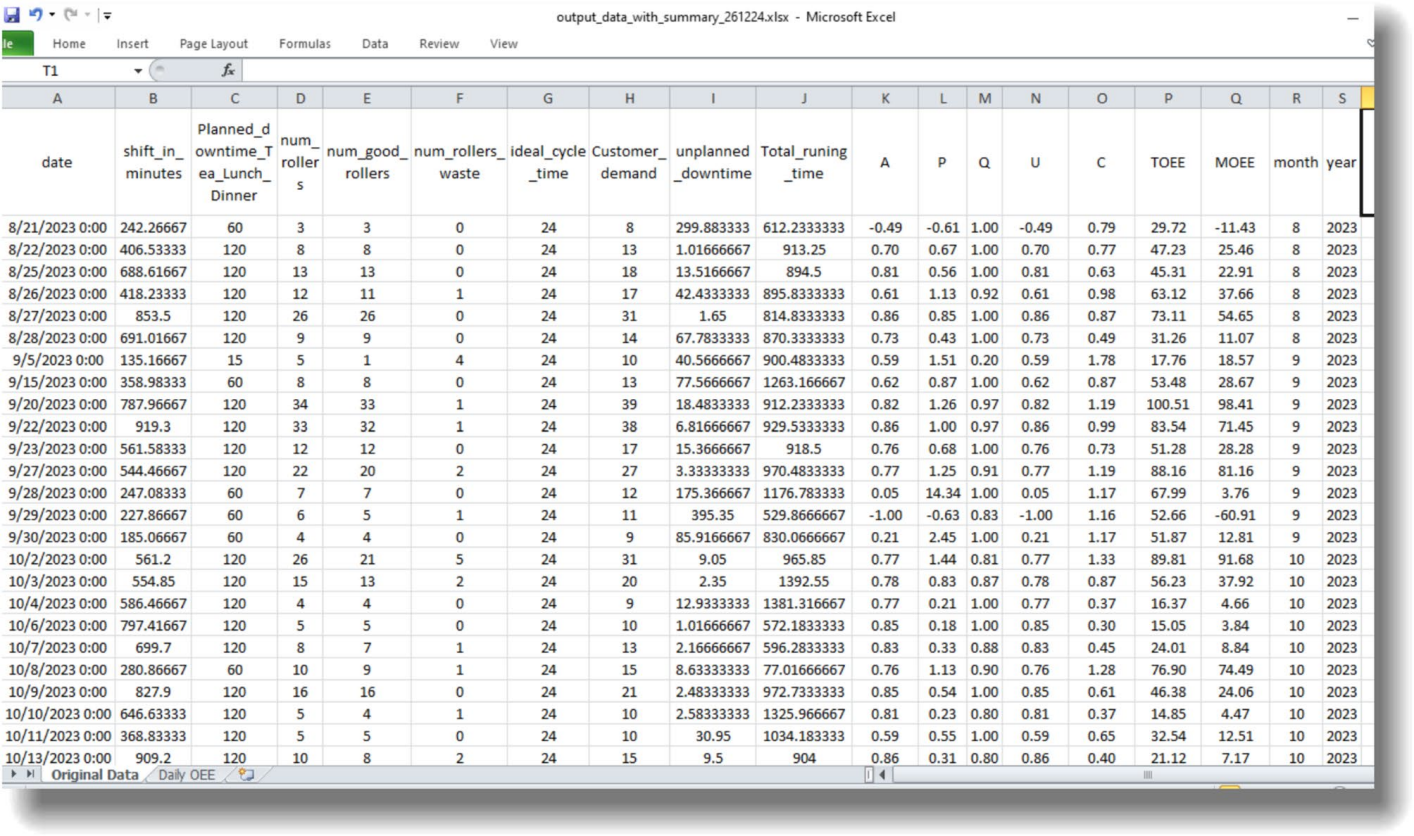
MATLAB Results - Comprehensive Output Data with Summary.

Figure 8: MATLAB generated ‘Output data with Summary’ document shows the results in a more thorough efficiency measurement. Such periods indicate bottlenecks on specific days. The key observations included that MOEE deviated more from TOEE at the monthly level than in daily variations. These deviations were more noticeable in months with: Irregular downtime, unplanned maintenance schedules, and Inconsistent shift patterns. This suggests that MOEE is more precise in reflecting actual operational efficiency. TOEE may sometimes exaggerate performance due to its less detailed calculation method.

**Fig. 9:**
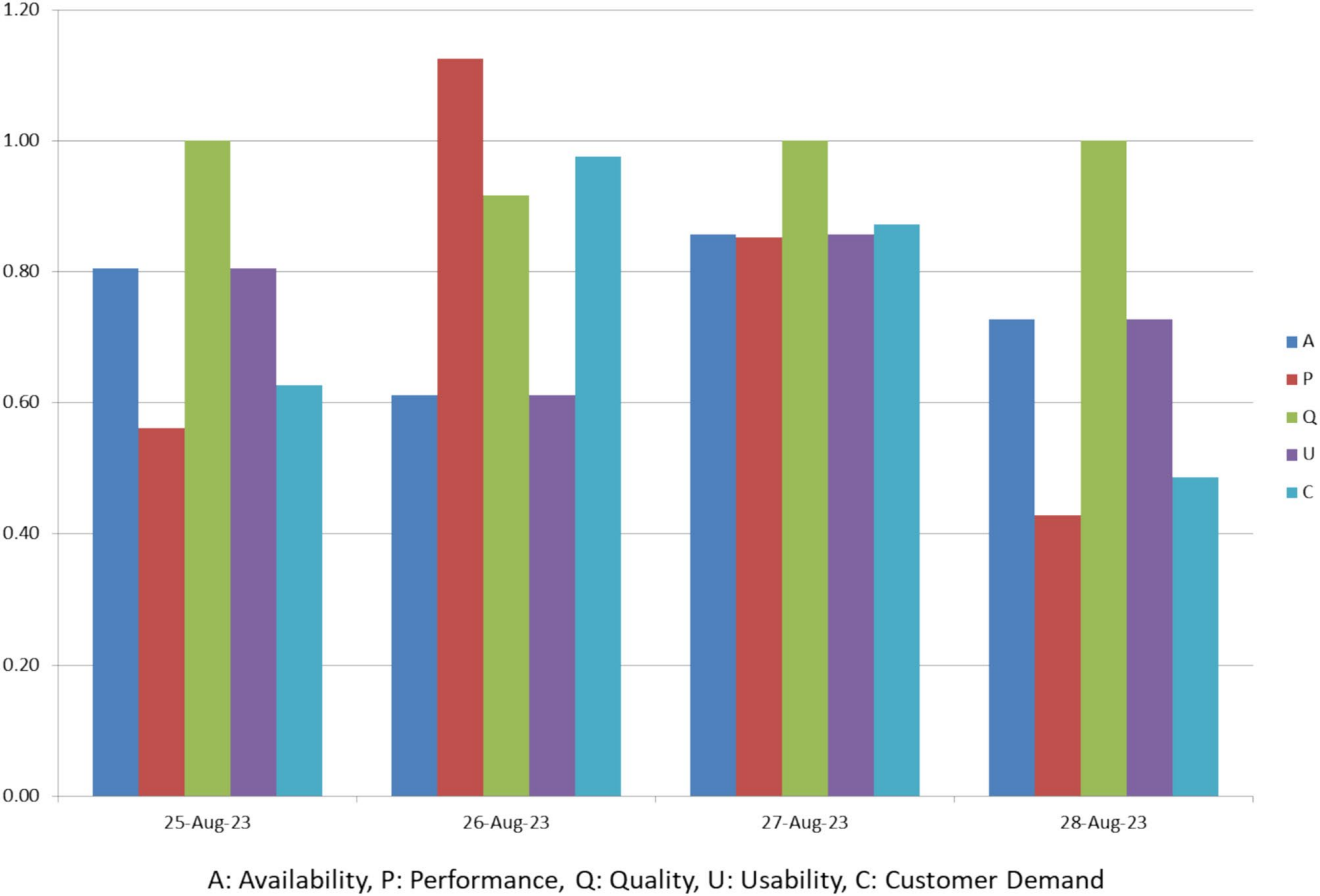
MOEE Parameter Analysis - Exploring Parameters ‘A, P, Q, U & C’.

Figure 9: MOEE Parameters – ‘A, P, Q, and U & C’ outlines the bar graph representation of MOEE parameters during the dates 25^th^ August 2020 till 28^th^ August 2023.

**Fig. 10:**
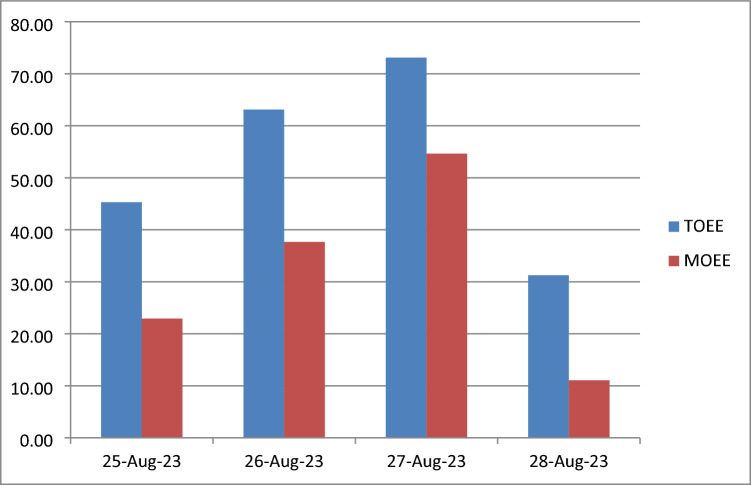
Comparative Assessment of TOEE and MOEE in Operational Efficiency.

**Fig. 11:**
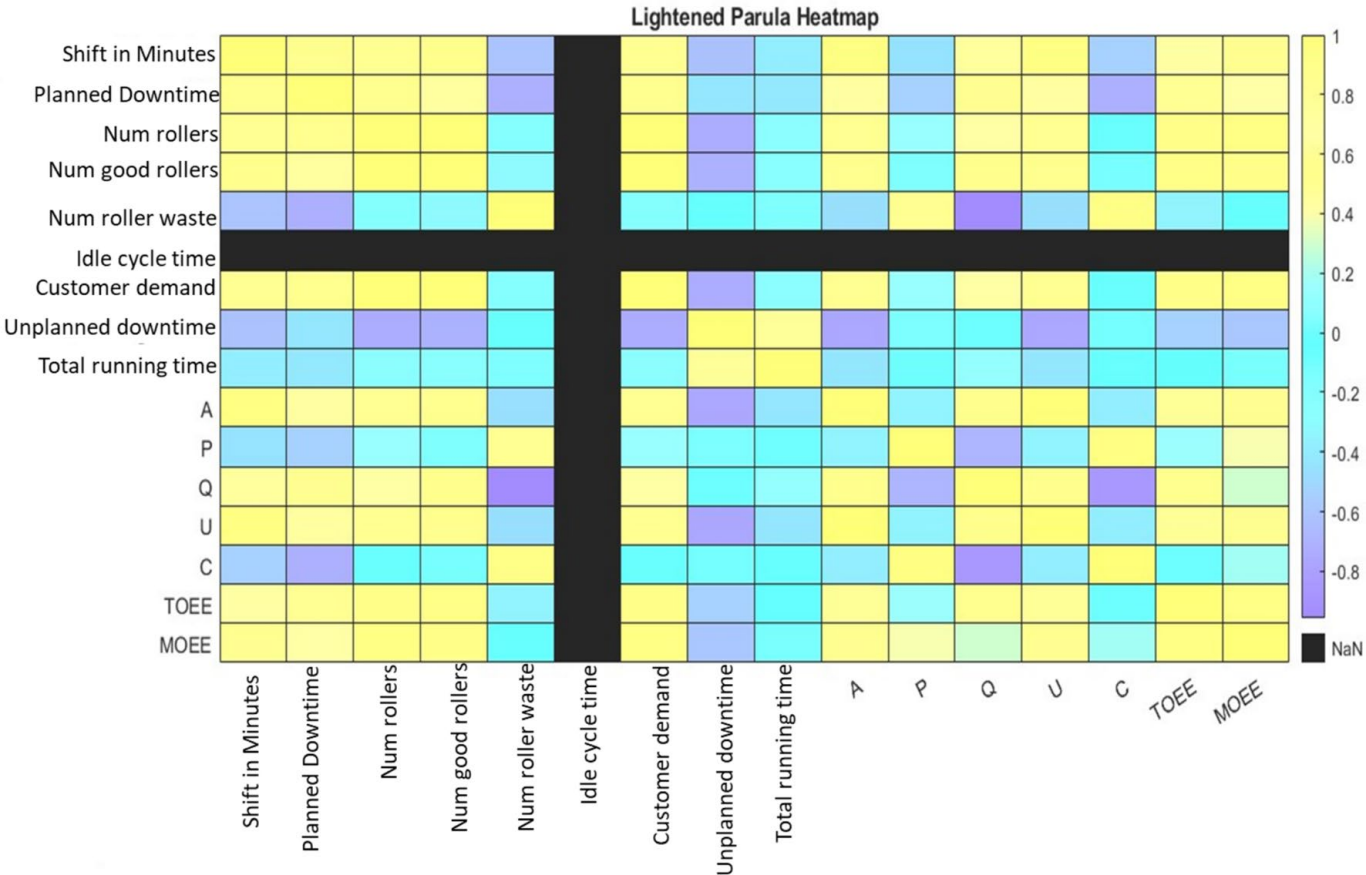
Heat Map of Operational Parameters.

Similarly, Figure 10: outlines comparative analysis between TOEE and MOEE For example, when downtime was high, MOEE dropped below TOEE, reflecting its significant impact on equipment performance. This comparison provided a more detailed understanding of equipment performance across: Multiple shifts, Different operational settings. Figure 11: Heat map outlines visualizing the performance data is crucial for identifying trends and anomalies in the production process. A heat map can help identify patterns in data, especially for large datasets, where the factors compared like customer demand and cycle time across months and shifts etc.

Table 4 Employee Shift Details and Downtime Allocation (Including Breaks) outlines information about the working shifts of company in hours as well as in minutes. In company, each employee must assign specific time slots for their breaks, such as tea breaks, lunch, and dinner. These set break times breaks are important to ensure a good work-life balance and to keep productivity levels high throughout the day. Employees are obligated to follow the break schedule to keep the workflow uninterrupted and avoid unnecessary interruptions. By adhering to these break times, everyone ensures they get adequate rest while keeping operations running effectively.

By examining a single entry from the Figure 8, as presented in Table 5, it becomes evident how the parameters ‘U’ and ‘C’ play a critical role in identifying machine’s potential to improve MOEE,

**Table 5:** Impact of Usability and Customer Demand on MOEE.

Date	Shift (Hr)	shift (Min)	Total Output	Waste	Total Goods	Ideal cycle time (Min)	Customer demand	*Running Time* (Min)	A (%)	P (%)	Q (%)	U (%)	C (%)	TOEE (%)	MOEE (%)	TEEP (%)	OEE (%)	**OFE (%)**
Machine parameters									Input parameters					Output parameters				
5-Sep 2023	10.1	610.2	10	2	8	23.7	25	488.2	80	49	80	80	97	31.10	24.1	25	38.8	31.36

The Usability ‘U’ value of 0.80 indicates that 80% of the equipment’s potential was effectively used during the shift. This outlines that although the equipment was in operation for most of the time, there is still opportunity to improve its efficiency. Usability can be affected by factors like equipment dependability, ease of operation, and operator skill.

The Customer Requirement Rate (C) is represented by 0.97, showing that production is nearly matched with customer demand, with just a small gap. A high C value (close to 1) indicates that the production rate is properly matched to meet customer needs, which is crucial for customer satisfaction and maintaining competitive advantage. If C is too low, it may suggest that production is falling behind customer demand, causing delays, missed deadlines, or stock shortages.

Hence, MOEE is directly affected by both Usability ‘U’ and Customer Requirement Rate ‘C’. The MOEE value of 24.18% suggests that there is room for advancement. Improving usability can boost MOEE by reducing downtime and ensuring that the equipment functions at its full capacity.

Table 6, Comparing TOEE, MOEE and TEEP, TEEP by definition has to consider Usability parameter while computation and hence the results from TEEP is slightly but not much better than MOEE. As MOEE has considered 2 new parameters while computing the equipment’s efficiency, the value of MOEE is slightly low i.e. 0.91 is the difference between the proposed MOEE and existing TEEP. OTE (Overall Throughput Efficiency) is nothing but achieved actual output is compared with its theoretical maximum value, which is 38.80% greater than that of MOEE. OFE is Overall Functional Efficiency; it is Similar to OEE but often used to assess how the machine performs against its full functional design.

**Table 6:** OEE and Extended Metrics Performance Summary.

Metric	Definition	Observation	Action required
TOEE	Machine is running during schedule time only	performance is low (only 49%), dragging OEE down	Improve speed, reduce small stops and idling
MOEE	Machine is running during schedule and unscheduled time with the customer demands	Utilization and customer demand impact the real efficiency	Maintain good utilization and demand alignment
TEEP	It shows how much output the machine gives compared to what it could produce if it ran 24/7.	achieving about 25% of total possible output	Focus on improving performance during scheduled time
OTE	It measures the throughput efficiency relative to total time	Often impacted by performance losses	Enhance production speed and minimize downtime
OFE	Functionally similar to OEE	Identifies machine’s functional capabilities and constraints	Improve maintenance and optimize operational performance

This was the comparison of all extended metrics performances. The corrective action after the overall monitoring and tracking was to decrease the waste from ex-06. Till 2023, no such real-time instrument was present in current company to maintain the record of waste; the operator manually collected and weighted the wastes. But as the company implemented a new IoT based system that enables real-time monitoring and recording of waste per machine.

**Fig. 12:**
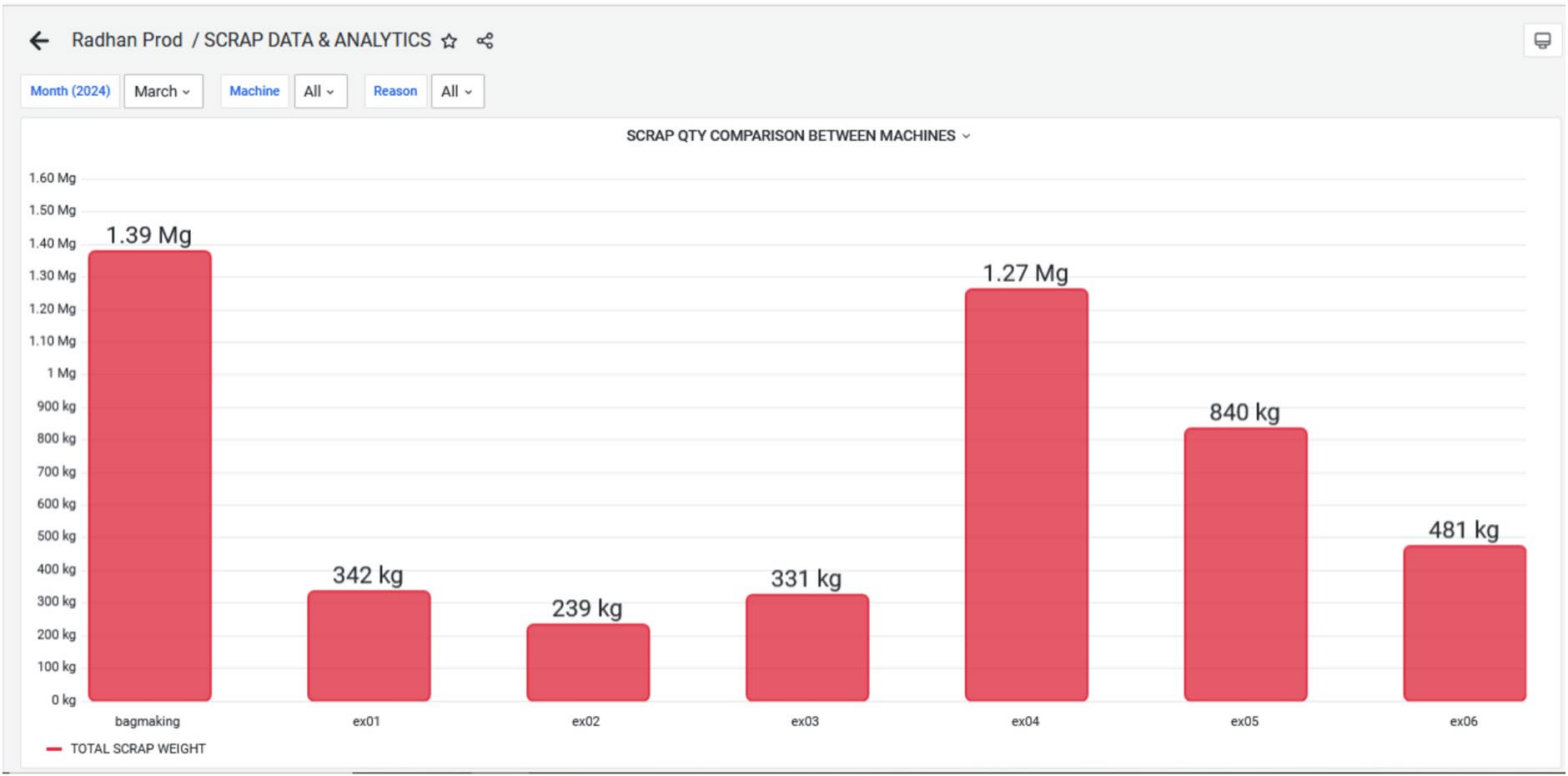
Scrap Data & Analytics for March 2024, highlighting the Volume and Categories of Scrap generated during Production.

**Fig. 13:**
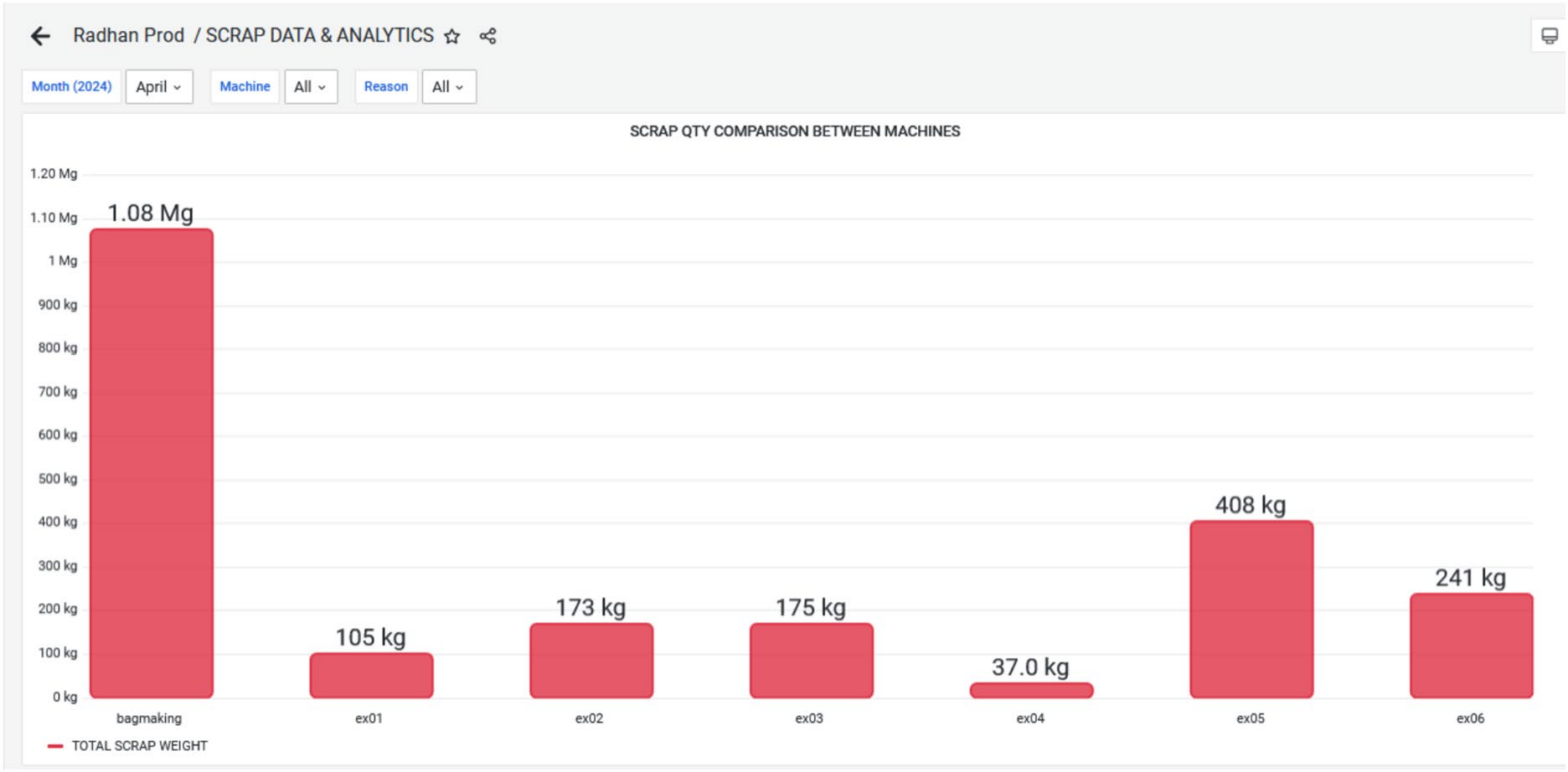
Scrap Data & Analytics for April 2024, showing Real-Time Monitoring of Defective Units, enabling Early Detection of Inefficiencies and Supporting Data-Driven Waste Reduction Strategies.

The dashboard Figure 12 now displays the graphical view of waste generated from each extrusion machine. This leads to better transparency and decision-making. This was not possible with their earlier manual system. Figure 13 demonstrates the reduced waste count especially the targeted plastic extruder machine ex-06. During March 2024, the wastes are 481 kg and it has reduced to 241 kg in the next month i.e. April 2024. This overcome was a great success towards reducing the waste, and reusing the waste for other purposes. The cost of raw material ranges from 30–100 kg depending on the material. If consider the material used is of LLDPE, then cost of the material 50 rupees per kg. By considering this the waste during March till April has come down to 240 kgs. Still the company has 20k rupees loss per month.

**Fig. 14:**
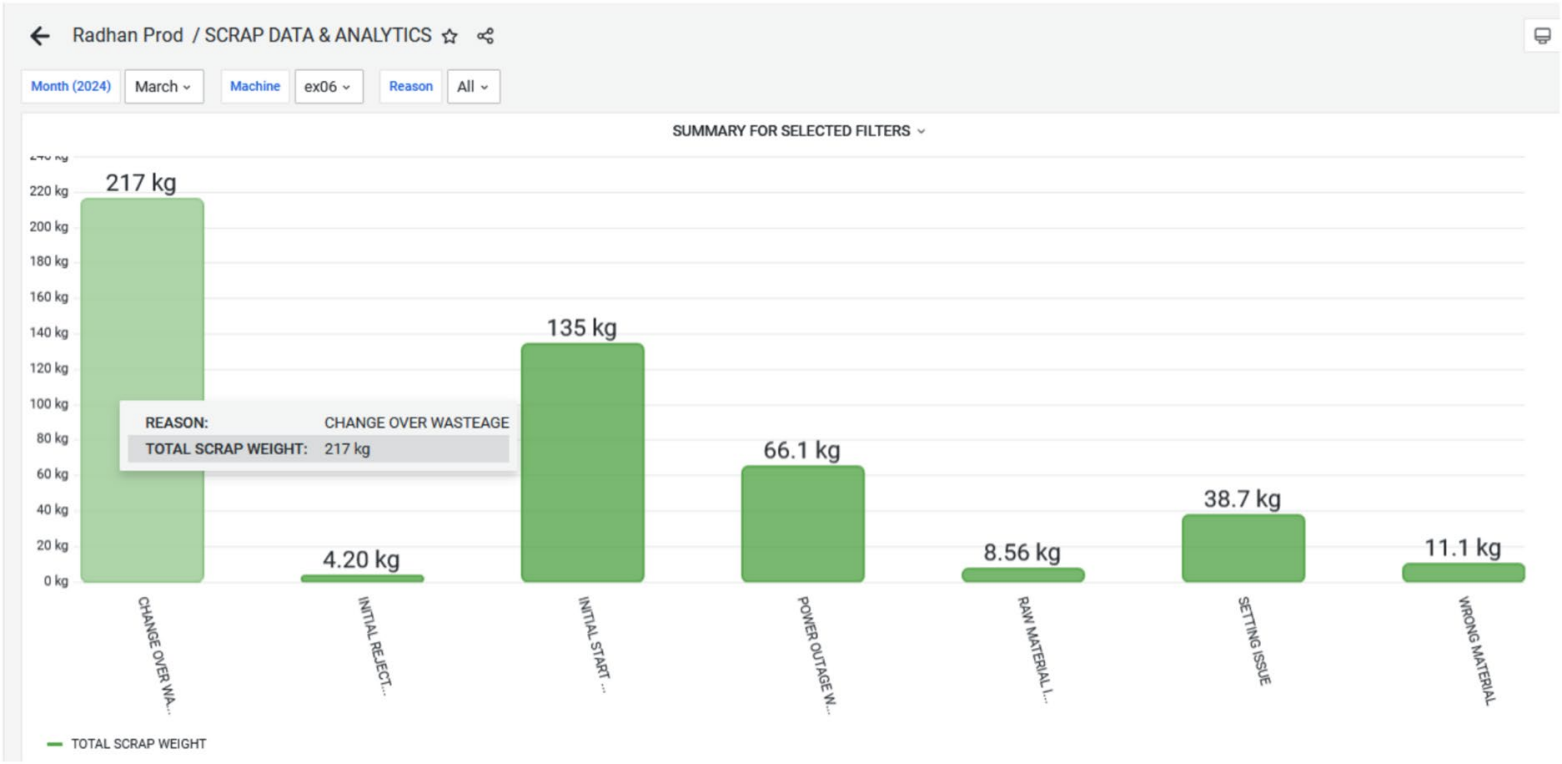
Monthly Wastage Summary for March 2024, showing the Distribution of Material Losses across Different Production Stages.

**Fig. 15:**
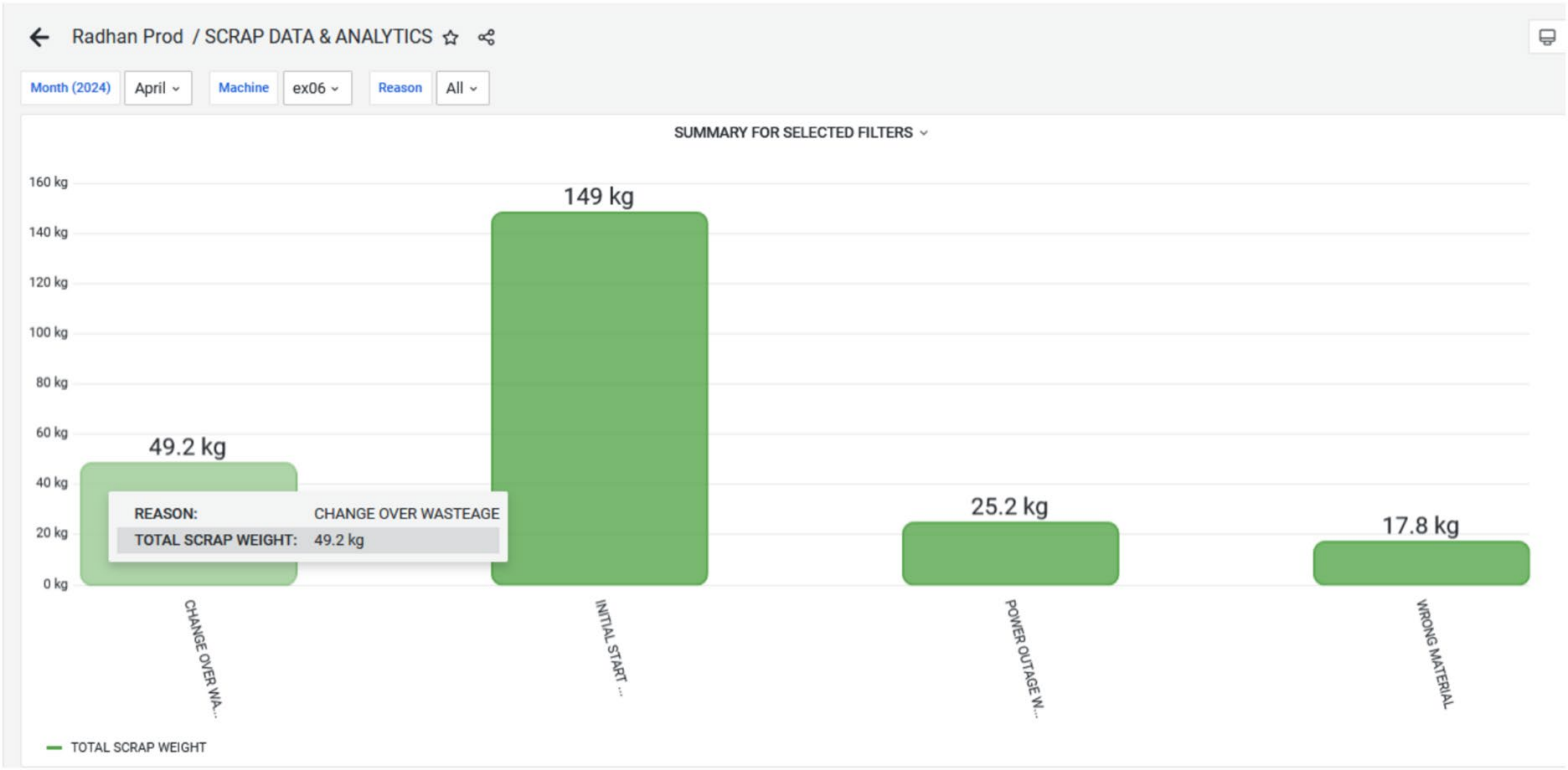
Summary of Material Wastages for April 2024, Indicating the Major Contributing Categories.

Figure 14 and Figure 15 elaborates, During March and April 2024, various categories of waste were recorded for the EX-06 machine, including changeover wastage, initial rejection; initial start wastage, power outage wastage, raw material issues, setting issues, and wrong material usage. In March, the highest waste was from changeover activities (217 kg), followed by initial start wastage (135 kg) and power outage-related losses (66.1 kg). Other contributors included setting issues (38.7 kg), raw material problems (8.56 kg), wrong material (11.1 kg), and minimal initial rejection (4.20 kg). In contrast, April showed significant improvements in several areas. Changeover wastage dropped to 49.2 kg, and initial start wastage, raw material issues, and setting issues were completely eliminated. However, initial rejection waste surged to 149 kg, indicating possible quality concerns during that period. Power outage wastage also reduced to 25.2 kg, while wrong material usage slightly increased to 17.8 kg. Overall, while April saw notable reductions in operational and material-related losses, the sharp rise in initial rejections suggests a need for improved quality control or operator training.

The Table 7 outlines the losses observed during dates 25^th^ August till 28^th^ August 2023. It also outlines the contributing factors to the losses and corrective actions. On 25^th^ August 2023, machine breakdown and setup/changeover losses were addressed through preventive maintenance and reducing setup time. On 26^th^ August 2023, minor stoppages and setup losses were tackled by improving training, adjusting processes, and optimizing setup intervals. On 27^th^ August 2023, the focus was on reducing setup/changeover frequency and improving defect prevention to minimize errors. On 28^th^ August 2023, remedial measures aimed at root-cause analysis and streamlining changeover procedures were implemented to address poor equipment availability and excessive setup time.

**Table 7:** Equipment Efficiency and Loss Analysis Report.

**Date (DD-M-23)**	**TOEE (%)**	**MOEE (%)**	**Loss Detected**	**Parameter Responsible for Loss**	**Corrective Actions**
25-Aug-23	45.31	22.91	Equipment Failure, Setup & Changeover Losses	Availability (Machine Breakdown)	Implement regular preventive maintenance and focus on reducing setup/changeover time.
26-Aug-23	63.12	37.66	Minor Stoppages, Setup & Changeover Losses	Performance (Minor Stoppage, Reduced Speed)	Reduce minor stoppages through better training or process adjustments, and optimize setup/changeover frequency.
27-Aug-23	73.11	54.65	Setup/Changeover Losses, Defects	Usability (Frequency of Setup Process), Quality (Defects)	Focus on reducing frequency of setup/changeovers and improving changeover process efficiency. Improve quality control to reduce defects.
28-Aug-23	31.26	11.07	Poor Equipment Availability, Excessive Setup Time	Availability (Equipment Failure), Usability (Excessive Setup Time)	Perform root cause analysis on machine failures, streamline setup processes, and minimize changeover time.

These findings demonstrate that integrating MOEE with IoT-based systems can provide industries with real-time monitoring and actionable insights, thereby enabling process optimization and supporting data-driven decision-making in manufacturing operations.

## Conclusions

The analysis examined TOEE and MOEE at both daily and monthly levels. This analysis revealed important insights into the manufacturing system’s operational performance. While TOEE provides an overall efficiency snapshot, MOEE incorporates additional variables that better reflect the true operational challenges faced during production. Key findings include ‘Instances where MOEE was consistently lower than TOEE, indicating significant downtime or inefficiencies’, and ‘The superior reliability of MOEE in predicting performance, especially during periods of irregular operational patterns’. Several operational factors influenced TOEE and MOEE. Key elements affecting these metrics included shift time, downtime, number of rollers, and maintenance schedules. The combination of these factors in MOEE provided a deeper understanding of the manufacturing process. MOEE captured more refined performance variations compared to TOEE. TOEE and MOEE evaluated in different manufacturing scenarios.

TOEE was useful for monitoring general equipment performance. MOEE provided a more trustworthy measure of operational efficiency. MOEE was particularly effective in environments with high fluctuations in: Shift patterns, Downtime, Machine maintenance. MOEE incorporated additional operational factors, such as: Number of rollers, Shift times, Impact of downtime. This enhanced MOEE to better predict overall efficiency. MOEE outperformed TOEE in identifying inefficiency periods from a predictive perspective. MOEE’s ability to adjust for downtime made it a more reliable reflection of the system’s real performance level.

This study contributes to the understanding of operational efficiency metrics by:

- Demonstrating that MOEE, with its more complex formula, offers a more accurate and nuanced reflection of equipment efficiency compared to TOEE.
- Highlighting the operational factors such as downtime, shift time, and roller utilization that impact these metrics, providing a foundation for improving production processes.

MOEE provides a more accurate and dynamic measure of manufacturing efficiency by incorporating real-time operational data. Its broader impact lies in enabling manufacturers across industries to optimize processes, improve productivity, and support data-driven decision-making. MOEE, therefore, serves as a more effective tool in measuring and improving operational efficiency, offering a robust approach for manufacturers to pinpoint inefficiencies and implement corrective measures.

## Future work

A company with annual turnover between 5–25 Cr. falls into small scale industry in India. Specifically this turnover aligns with the definition of small enterprise with MSME framework. The initial cost of implementing IoT, sensors, and related technologies may be high, but afterward, only maintenance is required. MOEE offers improved granularity in performance measurement. However, it relies heavily on real-time demand data and IoT-based inputs. This dependency may limit its practicality for SMEs operating with legacy systems or minimal digital infrastructure.

During the evaluation of MOEE, several limitations were observed. It was found that the approach is highly dependent on accurate and real-time data inputs, such as downtime records, shift timings, and maintenance logs. However, in resource-constrained manufacturing setups, such data may not be consistently available or automatically recorded. In such cases, a streamlined or minimal version of MOEE could be more practical and applicable. This study showed that MOEE can improve forecasting accuracy under certain operating conditions. Yet, some important factors were not included in the model. These include machine age, external environmental influences, and unexpected process disturbances. Such variables can have a significant impact on efficiency outcomes. Hence, future studies should focus on adding these variables to make the analysis more robust and comprehensive. Furthermore, by integrating real-time analytics and predictive modeling, the system’s adaptability and reliability cab be further improved. These advancements can be especially beneficial in dynamic manufacturing environments, where conditions frequently change. Theses directions for future work based on the data gaps and limitations identified during the analysis phase.

Future research could focus on developing simplified or lightweight versions of MOEE. Such adaptations would aim to make the approach more feasible for low-resource manufacturing environments. Despite the valuable insights offered by the analysis, several limitations were noted, the dependency on accurate and real-time data for downtime, shift times, and maintenance schedules and the simplification of certain factors that may further influence efficiency, such as the condition of machinery or external production constraints. Future work should focus on incorporating additional variables such as machine age, external environmental factors, and detailed machine-specific performance data. Additionally, integrating real-time data analytics and predictive modeling could further enhance the reliability of both TOEE and MOEE in dynamic manufacturing environments. Although the real-time dataset used in this study comes from a single machine a ‘Plastic Extruder’ using only a temperature sensor, the proposed approach can still be applicable to other manufacturing environments with different types of sensors. The main variation would occur during the data preprocessing stage.

## Data Availability

The dataset used in this study is sourced from Radhan Plastics, a company established in 2008, specializing in manufacturing films, tubing, bags, and covers from materials like EVA, VCI, Bubble, and LDPE. Their products serve industries including rubber compounding, pharmaceuticals, food, agriculture, packaging, and automotive. Located in Pirangut, near Pune, India, Radhan Plastics leverages Industry 4.0 technologies such as smart automation, real-time monitoring, and data-driven production processes. Due to privacy concerns and third-party data sharing agreements, the dataset is not publicly available. However, access to the data may be granted upon reasonable request to the corresponding author, subject to applicable conditions and approvals. First Author: Pranita Bhosale Email: pranitatambe@aitpune.edu.in Phone: +91-9545003009 Institution/Organization: Army Institute of Technology Dighi Hills, Pune, India

## Abbreviations

OEE: Overall Equipment Effectiveness
TOEE: Total Overall Equipment Effectiveness
MOEE: Machine Overall Equipment Effectiveness
A: Availability
P: Performance
Q: Quality
U: Usability
C: Customer Requirement Rate
Min: Minutes (time basis)
TEEP: Total Effective Equipment Performance
OTE: Overall Throughput Effectiveness
OFE: Overall Factory Effectiveness
